# Needs, resources, and barriers among family caregivers of people with dementia: a qualitative study

**DOI:** 10.1007/s40520-026-03436-x

**Published:** 2026-07-03

**Authors:** Chiara Milani, Claudia Biagi, Claudia Rosi, Rachele Mei, Fiorenza Wetzell Cabrera, Diletta Buresta, Marco Del Riccio, Riccardo Barucci, Enrico Benvenuti, Lorenzo Baggiani, Giulia Naldini, Chiara Lorini, Guglielmo Bonaccorsi

**Affiliations:** 1 Department of Health Sciences, University of Florence, Florence, Italy; 2 Geriatric Unit, Azienda USL Toscana Centro, Florence, Italy; 3 Department of Community Healthcare Network, Azienda USL Toscana Centro, Florence, Italy

**Keywords:** Dementia, Caregiving burden, Health literacy, Qualitative method, Primary care

## Abstract

**Background:**

Dementia poses major challenges for public health and care systems, with family caregivers serving as the primary source of support.

**Aims:**

This study explored the perceived needs, resources, and meanings attributed to the caregiving experience by family caregivers of people with dementia in a community-based setting.

**Methods:**

An exploratory qualitative study was conducted using individual semi-structured interviews at a community health center. Sociodemographic and care-related information was collected, and four standardized questionnaires were administered for descriptive contextual purposes only. Qualitative data were analysed thematically with a two-phase approach, starting from an existing framework on meaning-making and using a secondary categorization for defining a conceptual model of barriers and resources.

**Results:**

Twenty-five caregivers participated in the study. Levels of psychological distress and caregiver burden indicated emotional strain. Qualitative analysis identified three main themes and seven subthemes: (1) Complicated demands and healthcare interactions, including management of complex care needs and interaction with services; (2) Familial and relational dynamics, encompassing changes in family and social relationships, and shifts in reciprocity and role redefinition; and (3) Coping strategies and identity transformation, involving care as a source of personal meaning, sense of caregiving competence, and personal transformation. A conceptual model highlighted interconnected barriers and resources across individual, relational, and structural levels.

**Discussion:**

The identified themes advance understanding of caregivers’ lived experiences and the interplay between individual, relational, and structural factors.

**Conclusions:**

The study highlights the importance of considering caregivers’ coping strategies, health literacy, and resources through dedicated community-based interventions aimed at promoting their well-being and enhancing dementia care sustainability.

**Supplementary Information:**

The online version contains supplementary material available at https://doi.org/10.1007/s40520-026-03436-x.

## Introduction

The number of people living with dementia is expected to rise from 55 million in 2019 to 139 million in 2050, according to the World Health Organization (WHO), posing major challenges to public health and healthcare systems worldwide [1].

Within this context, informal caregivers - individuals who provide care, assistance or support to relatives or significant others without financial remuneration - play a central role, as most people living with dementia depend primarily on family members or other unpaid informal caregivers for daily support [2]. Approximately 40% of dementia-related costs are covered by family care [3, 4]. As the disease evolves, caregiving often requires many hours of daily assistance over extended periods [5], generating stress that negatively affects caregivers’ physical [6, 7] and mental health [8, 9, 10]. Anxiety and depression symptoms are strongly associated with the perceived intensity of their role [11]. Caregivers also face financial strain [5] and reduced opportunities for self-care and healthy lifestyles [12].

Health literacy (HL), defined as the ability to access, understand, appraise, and use health information to make appropriate health decisions [13], may help caregivers identify and use available health and social care resources effectively.

The WHO promotes the development and implementation of support and training programs for caregivers [2]. Such interventions should consider familial and cultural context [14]. Moreover, the literature highlights that effective interventions should address not only practical and informational needs, but also the existential dimensions of caregiving. Indeed, caregivers’ interpretation of their experiences influences the quality of care provided, as well as their motivation and psychological well-being [15]. A sense of competence and strengthened emotional bond can enhance caregiving satisfaction. Therefore, alongside the analysis of risk factors, it is also important to investigate the positive outcomes of the caregiving experience to inform the development of more effective interventions [16].

Despite the increasing recognition of their importance, informal caregivers often face isolation, lack of support services, and insufficient institutional recognition [17] as co-leaders in the care process. Greater attention to their lived experience is needed to ensure that policies reflect their priorities and to better understand factors influencing access to care [18]. The literature further highlights the need for the exploration of caregivers’ experiences through person-centred interventions that are co-designed with caregivers and tailored to diverse family contexts [19, 20].

The present study aims to describe caregivers’ experiences of caregiving for people with dementia in a community-based setting, focusing on health-related needs, available and perceived resources and role changes. Specifically, It explores how caregivers interpret and give meaning to their role and changing relationships, and it identifies perceived barriers and resources to inform the development of supportive interventions in community-based settings that better address caregiver wellbeing.

## Methods

### Study design and setting

The study adopted an exploratory qualitative design and was conducted in a specific community health center, a House of Community (HoC; “*Casa della Comunità*”) in the city of Florence. This facility served as an experimental model for primary care research and development in Tuscany [21]. The present study is part of a broader project aimed at designing, implementing and evaluating innovative group-based interventions for caregivers of people with dementia at the community level.

Ethical approval was obtained by the Ethics Committee of the Tuscany Region – *sezione Area Vasta Centro*,* on 17.05.2024*,* version 1st*.

The reporting of this qualitative study followed the Standards for Reporting Qualitative Research (SRQR) [22]. The SRQR checklist is provided as *Supplementary file 1*.

### Participants selection and recruitment procedures

Participants were eligible if they were adult informal caregivers of a person with dementia, defined as non-professional individuals providing regular, unpaid care, including family members or individuals in a close, family-like relationship. Participants were required to express their willingness to participate in a subsequent support group program, and to provide written informed consent, after receiving study information from the research team. Adequate Italian language proficiency was required. Participants were recruited through convenience sampling.

Despite the broader eligibility criteria, the final sample consisted exclusively of 25 family caregivers which may emphasize relational and family-related themes in the findings. Recruitment and interviews took place between April 2024 and May 2025, supported by health and social care professionals (e.g., geriatricians, general practitioners, social workers, and nurses).

### Data collection

Data were collected using multiple sources, with semi-structured interviews (SSIs) representing the primary data source. They were conducted using an interview guide developed by the research team (Table 1) and informed by previous literature [23] to explore caregivers’ perceived resources and barriers at the individual, family, and social levels.

**Table 1:** Overview of the Semi-Structured Interview guide for caregivers

Information and understanding of the illness
● What information have you received regarding your family member’s illness and its progression? ● From whom did you primarily obtain information about the course of the disease? To what extent did you find this information sufficient for developing a clear understanding? ● How would you describe the current understanding of the progression of your family member’s illness? ● What changes do you anticipate may be necessary in the future? How do you feel about these potential changes?
**Relationship with services**
● To what extent do you feel you have received adequate support from health and social care services? ● Which services did you find most supportive and which did you find less supportive? ● How would you describe and evaluate your relationship with these services over time?
**Impact of illness on the caregiver’s life**
● Since the diagnosis, how has your social life changed (e.g., work, friendships, hobbies)? ● What changes have you experienced in terms of your personal self-care and personal well-being? ● How has the illness affected the management of your home and family life?
**Relational and family dynamics**
● In what ways do you feel the diagnosis affected your relationship with your family member? ● What were the family’s initial reactions to the diagnosis? ● How have family relationships changed following the diagnosis of dementia? ● How would you describe the current family climate? ● Do you feel that there are people close to you, within your family or outside, on whom you can rely?
**Emotional Experiences**
● How did you react to your family member’s diagnosis of dementia? ● How do you feel you have adapted to your caring role? ● What emotions are most frequent and in which situations? ● To what extent do you feel able to manage them? In what way?

In addition, participants completed a short questionnaire collecting sociodemographic information and care-related information. Considering caregiver’s sociodemographic characteristics, variables collected included age (years), gender, citizenship, educational level (primary school certificate, lower secondary school diploma, upper secondary school diploma, bachelor’s/master’s degree), employment status (employed, unemployed, retired, homemaker), marital status (married/cohabiting, single, widowed, divorced/separated), and perceived economic condition. The latter was assessed by asking participants how easily they manage to make ends meet with their available financial resources, with 4 response options ranging from “very easily” to “with a lot of difficulty). Data concerning the person with dementia included the caregiver’s relationship to the family member with dementia, the person’s age, year of diagnosis, length of caregiving role, daily care demands, and the number of individuals involved in providing care.

Standardised tests were used to descriptively characterize participants and to contextualize the qualitative findings; given the exploratory nature of the study, these measures were used for descriptive purposes rather than for inferential statistical analysis. Specifically, we assessed well-being using the World Health Organization-Five Well-Being Index (WHO-5) [24], HL using the Italian version of the HLS-EU-Q16 [25], symptoms of depression, anxiety and stress using the Depression, Anxiety, Stress Scales (DASS-21) [26, 27], and caregiver burden using the Caregiver Burden Inventory (CBI) [28, 29]. These instruments were selected based on their widespread use in caregiving and dementia research and their ability to capture complementary dimensions of the caregiving experience [30, 31, 32, 33]. Internal consistency coefficients in the present sample were as follows: CBI α = 0.92; DASS-21 α = 0.93; WHO-5 α = 0.89; HLS-EU-Q16 α = 0.66. Further details of these instruments are provided in *Box 1*.

Box 1. Overview of standardized instruments administered to describe participants’ psychological well-being, health literacy, psychological distress, and caregiver burden. **Well-being**

It was assessed using the World Health Organization-Five Well-Being Index (WHO-5) [24], a validated short self-report instrument designed to measure subjective psychological well-being over the previous two weeks. Total score ranges from 0 to 25, with higher scores indicating better well-being. In line with established recommendations, a cut-off score of 13 suggests reduced well-being and the potential need for further clinical assessment.

**Health Literacy**

It was assessed using the Italian version of the HLS-EU-Q16 [25], a validated 16-item short form of the European Health Literacy Survey Questionnaire (HLS-EU-Q47). The items explore the perceived ease of accessing, understanding, appraising, and applying health-related information, and are rated on a four-point Likert scale from ‘very easy’ to ‘very difficult’. Total score, ranging from 0 to 16, is categorized into three levels: inadequate (0–8), problematic (9–12), and adequate (13–16) HL.

**Depression, Anxiety and stress**

Psychological distress was measured using the Depression, Anxiety, Stress Scales – 21 items (DASS-21) [26, 27], a self-report questionnaire comprising three subscales- Depression, Anxiety, and Stress- each consisting of 7 items. Subscale scores are obtained by doubling the raw scores of the Italian version, with values from 0 to 42, and with higher scores indicating greater symptom severity. Each subscale can be classified into five severity categories [26]: depression (0–9 normal, 10–13 mild, 14–20 moderate, 21–27 severe, and > = 28 extremely severe); anxiety (0–7 normal, 8–9 mild, 10 to 14 moderate, 15 to 19 severe, and 20 or above extremely severe; stress (0 to 14 normal, 15 to 18 mild, 19 to 25 moderate, 26 to 33 severe, and 34 or above extremely severe). These cut-offs provide a clinically meaningful framework for interpreting individual scores, also considering reference data from the Italian general population mean (M = 12.3, SD = 8.3) [27].

**Caregiver burden**

It was assessed using the Caregiver Burden Inventory (CBI) [28], a multidimensional self-report tool developed specifically for caregivers and extensively used in caregivers of people with dementia to assess the multidimensional burden of care. The CBI measures burden across five domains: time-dependence, developmental, physical, social and emotional burden. The total score ranges from 0 to 100, with higher scores reflecting greater perceived burden. As the CBI does not have universally accepted cut-off values, interpretation is generally based on group comparisons or reference values reported in the literature; for example, a mean score of 32.5 (SD = 18.0) has been reported for caregivers of people with dementia [29].

An individual meeting was scheduled in a private outpatient room at the HoC. Each meeting was conducted by two researchers (C.R. and C.B.), both trained in qualitative research methods, and lasted on average 60 min (range: 45–75 min). The participant first completed the questionnaires and then took part in the individual SSI, conducted in Italian language by one researcher who interacted directly with the caregiver, while the second produced a detailed written transcript of the conversation in real time. The researchers had no prior clinical or personal relationship with participants. To enhance trustworthiness, investigator triangulation and participant transcript verification were adopted during data collection. Specifically, investigator triangulation involved the presence of two researchers during each interview and the joint review of transcripts immediately after the interview, allowing comparison of observations, clarification of unclear passages, and discussion of preliminary interpretations. The transcript was then shared with the participant to verify its accuracy and completeness and to confirm that it adequately captured the content and meaning of the interview. Because the interviews were not audio-recorded, the written transcript should not be considered a verbatim transcript, but rather as a contemporaneous reconstruction of participant’s narrative. Data collection continued until thematic saturation was reached, defined as the point at which no new themes emerged from subsequent interviews.

### Data analysis

All collected data were anonymized by assigning a unique code and processed in anonymous form. Furthermore, any identifiable information originally present in the SSIs was either removed or anonymized.

Given the sample size, quantitative questionnaire scores (DASS-21, WHO-5, CBI, HLS-EU-Q16), along with socio-demographic variables, were summarized using descriptive statistics only (mean and ranges), without inferential analyses, to provide additional context to the findings from the SSIs.

Qualitative data analysis followed a two-phase process combining deductive and inductive approaches [34], with the aim of both engaging with and extending an existing theoretical framework.

In the 1st phase, we adopted a deductive theory-driven, yet flexible coding approach, using the framework proposed by Chen et al. on the meaning-making of dementia caregiving [15], as a sensitizing device rather than a fixed coding template. The three key dimensions of the framework − (1) complicated demands and customized care, (2) evolving dynamics in family and dual relationships, and (3) coping strategies - were used to guide the initial reading of the SSIs and to support preliminary code generation. The subsequent phases of the analysis were as follows: (i) two researchers (C.M. and C.R.) independently conducted open coding within and across these dimensions; (ii) within each dimension, codes were then inductively refined and grouped, allowing the existing themes and sub-themes to be adapted, merged, renamed or newly created; (iii) discrepancies were discussed and resolved through team-based meetings involving a third researcher, with particular attention to divergent interpretations; (iv) relationships among themes, sub-themes and codes were iteratively elaborated and refined and visualised in an evolving inductive coding table (see *Supplementary file 2*) .

In the 2nd phase, we conducted a secondary interpretative analysis of the codes. Each code was re-examined in its narrative context to better understand how caregivers described and gave meaning to their experiences. Specifically, codes were interpreted as either enablers/resources (positive value) or as barriers (negative value), acknowledging that similar experiences could assume different meanings depending on the individual and relational context.

Through iterative comparison of these barrier/resource categorizations, we developed a conceptual model organized across three interconnected levels:

- *micro* – individual dimensions, such as emotional resources, motivation, and personal agency,
- *meso* – relational and interpersonal level, such as family relationships, interpersonal dynamics, social networks, and.
- *macro* – structural level, which means organization of services, policies, access to services. The model was built through iterative comparison of the barrier/resource categorization.

The model emerged through an iterative analytic process, in which categorizations were continuously refined, contrasted across cases, and discussed within the research team, allowing the data to refine and extend the initial framework rather than simply illustrate it. Its micro, meso, macro structure was not derived from the original framework but emerged inductively from the iterative comparison of barrier/resource categorizations.

## Results

### Descriptive characteristics of the sample

This section presents the socio-demographic and caregiving characteristics of the twenty-five participants (Table 2). The sample comprised adult children (56%) and partners (44%) and was predominantly female (60%) and Italian (96%), with a mean age of 65 years (range 42–89). Most participants (88%) had up to upper secondary education, more than half (56%) were retired, 76% reported making ends meet easily. Regarding caregiving, 40% had been providing care for 5–10 years, 56% lived with the care recipient, and 28% reported receiving no support in caregiving.

With respect to standardized questionnaires administered for descriptive purposes, the mean score on the HLSEU-Q16 was 11.96 (range: 7–16), indicating a problematic level of HL. The mean score on the WHO-5 was 10.72 (range: 1–21), indicating relatively low levels of well-being, as scores below 13 are generally considered indicative of poor well-being. Psychological distress (DASS-21) showed a mean total raw score of 19.24 (range: 5–44), with mean subscale scores of 10.70 (depression), 17.42 (stress), and 7.29 (anxiety), suggesting mild to moderate levels of distress across domains. Finally, the mean CBI total score was 38.46 (range: 6.5–81), reflecting a moderate level of caregiver burden.

**Table 2:** Characteristics of the study participants (total sample = 25 subjects)

Characteristics	*N* (%) - Mean score (min-max)	Characteristics	*N* (%) - Mean score (min-max)
Gender Male Female	10 (40%) 15 (60%)	**Civil status** Married/cohabiting Divorced/separated Widowed Single	17 (68%) 4 (16%) 1 (4%) 3 (12%)
Citizenship Italian Foreign Age	24 (96%) 1 (4%) 65.36 (42–89)		
		**Relation to the care receiver/family member** Son/daughter Husband/wife/partner	14 (56%) 11 (44%)
		**Period of caregiving** < 1 year 1–5 years 5–10 years	1 (4%) 14 (56%) 10 (40%)
Educational level Primary school certificate Lower secondary school diploma Upper secondary school diploma Bachelor’s/master’s degree	2 (8%) 7 (28%) 13 (52%) 3 (12%)		
		**Living situation** Co-residing with family member Not co-residing with family member	14 (56%) 11 (44%)
		**Support in caring** No support Support from another caregiver (formal or informal) Service (day care or RSA) **WHO-5 score** **HLS-EU-Q16 score** **DASS-21 score** **CBI score**	7 (28%) 14 (56%) 4 (16%) 10.72 (1–21) 11.96 (7–16) 19.24 (5–44) 38.46 (6,5–81)
Perceived economic status Great difficulty Some difficulty Quite easily Very easily Employment status Employed Homemaker Retired Unemployed	3 (12%) 3 (12%) 12 (48%) 7 (28%) 8 (32%) 1 (4%) 14 (56%) 2 (8%)		

### SSIs’ findings

This section presents the findings from twenty-five SSIs with caregivers of individuals with dementia. We reorganized the data into three themes and seven sub-themes, adapting and renaming some of them.

In the 1st theme (“Complicated demands and healthcare interactions”), sub-themes 1 and 2 were merged into “managing complex care needs”. A new sub-theme, “interaction with services”, highlights the central role of healthcare systems in shaping caregiving experiences - an aspect that was less explicitly developed in the original framework. The 3rd theme was renamed “coping strategies”, to better capture the subjective and intrapersonal processes through which caregivers interpret and adapt to their role. This theme includes the following sub-themes: “identity changes and personal transformation”, “care as a source of personal meaning” and “perceived competence in caregiving”. All the themes identified were supported across a large proportion of participants (refer to Table 3), suggesting that they reflect shared experiences rather than being driven by a small number of individuals.

### Theme #1: complicated demands and healthcare interactions

The SSIs revealed that caregivers have to face complex health needs, which often are unmet without structured or institutional support. This theme focuses on the “practical and material aspects of caregiving”. Indeed, these needs are recognized as structural, objective, and material demands, often perceived as external to the caregivers’ personal willingness or control, highlighting that the burden is both a consequence of the severity of the disease and the gaps in formal support and service provision. Indeed, by positioning demands as external and structural rather than personal, it appears that caregivers try to define a narrative that preserves their identity as a meaning-making strategy.

#### 1.1 – Managing complex needs

Twenty-one caregivers (forty-eight quotes) reported that demands become increasingly complex, requiring a continuous process of adaptation, perceived as negative in thirty-seven quotes and generating the feeling of “always needing to be present” as interviewee n.2 affirmed: “*She depends on me for everything*,* I prepare her clothes*,* I cook her meals*,* I dress her. She is always with me*,* I can’t leave her*,* I don’t trust anyone else with her*”.

In some cases, the situation is so complex that it generates a perception of resource depletion: *“Everything is exhausting*,* I have to pay attention to so many aspects…it is draining”*, said interviewee n.24 and “*I feel completely absorbed”* (interviewee n.15).

Caregivers reported the need to identify specific solutions to adequately take care of health needs.

Interviewee n.15 described how he/she feels as follows: *“She gets confused at night*,* she doesn’t remember the way to the bathroom*,* so I lock the door at night to prevent her from leaving the house and possibly falling down the stairs again”.*

This sub-theme reveals a tension at the core of the caregiving experience: the progressive erosion of boundaries between self and role.

#### 1.2 – Interactions with services

Twenty-four SSIs referred to the presence and support of services as crucial, describing positive experiences with services that made them feel welcomed and accepted .

Interviewee n.1 stated: *“We had a very good experience*,* and the results were visible”.*

However, a sense of being invisible and not recognized as caregivers often emerged. Caregivers also described multiple barriers to access services – organizational, economic, communicational.

Interviewee n.10 affirmed: *“I found that there has not been enough support on how to handle daily life*,* how to deal with reactions. There is only the medical aspect*,* but in everyday life there is so much more…”.* And interviewee n.24: *“If my mother hadn’t had a daughter*,* who would have helped her? From my experience*,* institutions don’t help her…”.*

### Theme #2: Family and relational dynamics

The SSIs highlighted how relationships evolve due to the caregiving role and its increasingly complex and changing dynamics, showing how caregiving also modifies the distribution of support and burden within families and social networks. Indeed, caregivers continuously renegotiate the meaning of their relationships due to the fact that caregiving results in a reshaping of every interpersonal dynamic.

#### 2.1 – Changes in familiar and social relationships

Findings from nearly all semi-structured interviews (twenty-four) indicate that caregivers’ familiar and social relationships evolve together with the evolving of the care process and these relational changes are not uniformly positive or negative.

Caregivers reported that family relationships can be a significant source of support (twenty-nine quotes), but also of conflict and pain, especially when tensions arise (thirty-nine quotes).

Interviewee n.2 said: *“My brothers don’t realize the burden…”.*

Another participant (n.5) shared: *“Alzheimer drives friends away*,* they stop coming”.*

However, these changes can also lead to new forms of support where caregivers have to redefine their relational meanings and to interpret their relationships as part of adapting to their role. Interviewee n.1 affirmed: “*There is a bit more closeness*,* there is support between us…*”; while participant n.10 said: “*My daughter tries to support me*,* maybe she’s grown closer than before”.*

#### 2.2 – Shift in reciprocity and redefinition of role

Many SSIs revealed the dual relationship as becoming increasingly unilateral, with a loss of reciprocity, described as a negative feeling in sixty-six quotes.

Caregiver n.10 said: *“The problem is that you have a relationship with a person that at some point comes to end. He is no longer himself…”.*

This experience often leads to anticipatory grief and can be associated with a growing feeling of loneliness, as one interviewee echoed: *“I miss how it used to be. When I think about it*,* I feel sad”.*

However, some caregivers also highlighted their attempt to find new meanings, as follows: “*Since she has had this disease*,* she has made me feel useful to her - for the first and only time…”* (interviewee n.4).

### Theme #3: coping strategies

This theme focuses on the crucial role of the subjective process of elaboration and adaptation to the new situation and of the identity-related consequence of caregiving – focusing on the caregiver’s inner world: emotions, meanings, personal changes and transformations. It highlights how caregivers try to integrate the caregiving experience into their sense of self, their values and understanding. Negative cases suggest that the meaning in the role is found through difficulty and not in spite of it.

#### 3.1 – Identity changes and personal transformation

The SSIs revealed that caregiving is perceived as an ambivalent experience (eighty-nine quotes). It can be a source of pain and sacrifice due to the erosion of self, including the loss of personal time, interests, identity, social roles and emotional balance (sixty-nine quotes), as interviewee n.19 stated: *“I don’t do anything anymore because I don’t have the time*,* and now*,* I don’t even have the strength”.* Another participant (subject n.2) emphasized the ambivalence of the experience: *“I drag her around with me*,* and on the one hand*,* I’m glad because I know she’s with me*,* but it’s also a bit hard to always have her with me”.*

However, nineteen quotes also highlighted that caregivers could experience a process of personal growth, as interviewee n.7 described: *“The first month was really tough…but at the same time*,* that month shaped me*,* it made me stronger”.*

#### 3.2 – Care as a source of personal meaning

Caregivers described a process of reflection on their priorities and on the meaning of care in their lives. For some, this led to a re-evaluation of their value system, especially in relation to the concept of care. This process prompted them to reflect on their own activities and the values guiding their daily lives (twenty quotes). For instance, participant n.20 stated: *“Even the memory of what my father used to be*,* and the need I feel to give something back to him for the love I feel”.*

In this sense, care could become an important source of personal meaning and identity for some caregivers, alongside the burdens they experienced.

#### 3.3 – Perceived competence in caregiving

Caregivers report that the situation and difficulties of the caregiving role led to the development of a sense of competence, with confidence in their abilities and in managing challenges (thirty-six quotes).

As reported by participant n.24: “*I think that if she were able to socialize*,* she might open again and find some energy. Next week I will try to take her to the daycare center*”; interviewee n.16 stated: “*I’m looking for ways to buy some time and make sure this situation affects us as little as possible*”.

Otherwise, a poor sense of competence prevails, with the perception of being unable to cope with tasks and challenges of managing the situation (sixteen quotes). Participant n.23 stated “*There are moments when she is alone*,* and I don’t feel at ease. I feel anxious. It is hard for me to seek help*,* to have to find someone (…)*”; whereas participant n. 13 asserted, “*I feel powerless*,* I don’t know what to do*,* I wish things were a bit clearer*”.

All themes were represented in the data, although with different positive or negative connotations. Table 3 summarizes the distribution of quotes associated with each theme and sub-theme identified. Negative quotes predominated overall (257 out of 391 quotes), particularly within Themes 1 and 2. In contrast, Theme 3 showed a more balanced distribution, with a higher proportion of positive quotes, especially within sub-themes related to personal meaning and perceived competence.

**Table 3:** Distribution of themes and sub-themes across semi-structured interviews

Themes	Sub-themes	*N* of quotes/SSIs in which the topic is covered	*N* of quotes negative/ positive o ambivalent
#1 – Complicated demands and healthcare interactions	1.1 Managing complex needs 1.2 Interaction with services	48 quotes; 23 SSIs 45 quotes; 24 SSIs	37; 11 25; 20
#2 – Familiar and relational dynamics	2.1 Changes in familiar and social relationships 2.2 Shift in reciprocity and redefinition of roles	58 quotes; 24 SSIs 79 quotes; 24 SSIs	39; 19 66; 13
#3 – Coping strategy	3.1 Identity changes and personal transformation 3.2 Care as a source of personal meaning 3.3 Perceived competence in caregiving	89 quotes; 24 SSIs 20 quotes; 14 SSIs 52 quotes; 21 SSIs	69; 19 5; 15 16; 36
**Total**		391 quotes; 25 SSIs	257; 133

### Emerging conceptual model of barriers and resources

The qualitative findings were integrated into a conceptual model of barriers and resources within different levels of the caregiving process. At the micro level, the most represented, factors could be divided into: emotional regulations (where barriers primarily include feelings of inadequacy and emotional and psychological burden), the meaning assigned to caregiving (such as the ability to find meaning in daily routines and a sense of self-efficacy from caregiving) and the perceived competence with difficulties in self-care - which often limit caregivers’ ability to cope, but also with resources, such as adaptive coping strategies. At the meso level, the relational dimension emerged with a double meaning: the burden can be intensified by bad family dynamics, lack of support, and social isolation, but supportive relationships, effective communication, and positive relational histories can support resilience. At the macro level, factors were related to the caregivers’ interaction with the health and social care system. Frustration was linked with fragmentation and limited accessibility of services, and the perceived invisibility of their role, while continuity of care, institutional trust, and recognition of caregivers within care pathways were considered resources.

**Fig. 1:**
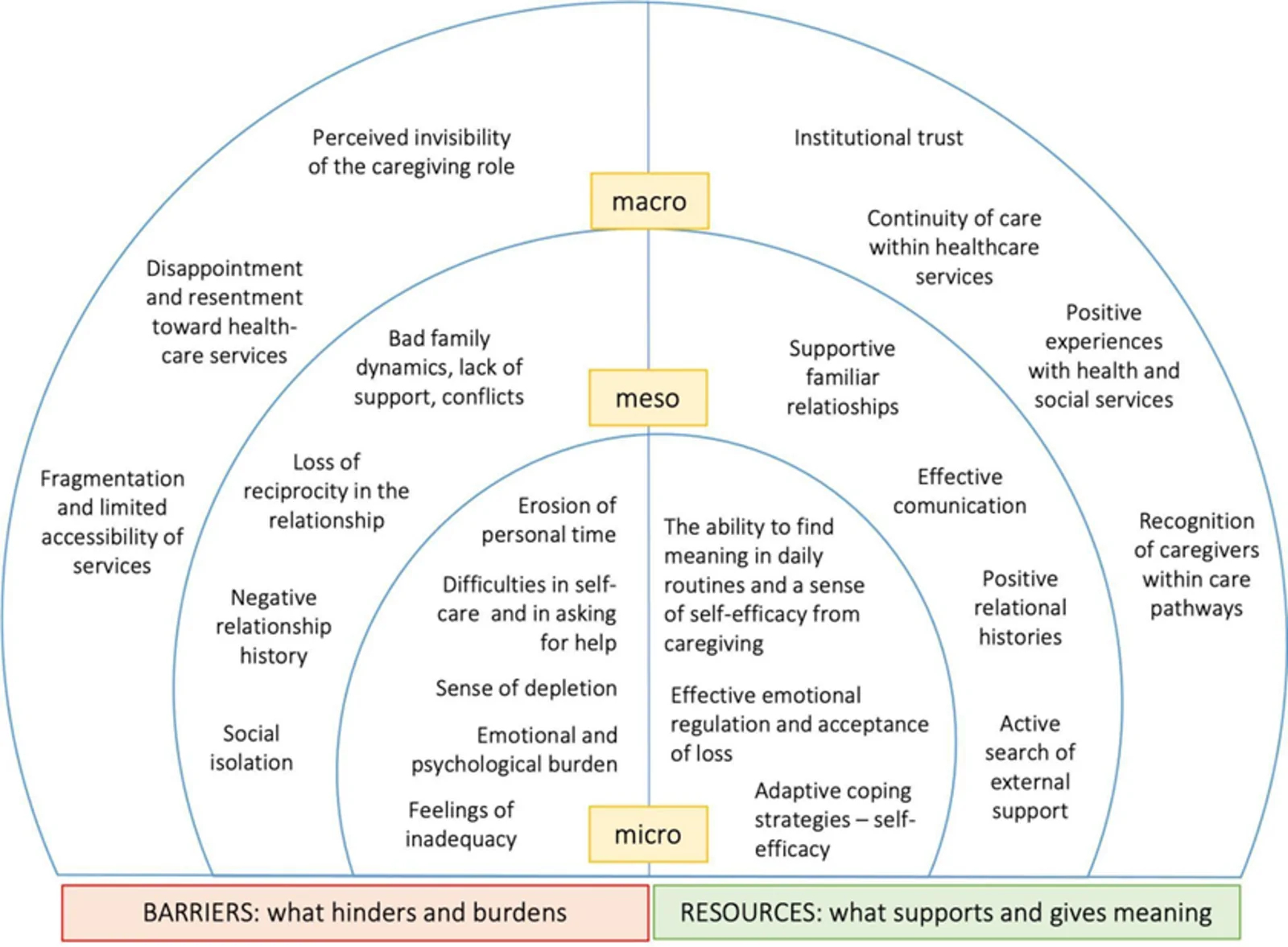
Model of barriers and resources

Overall, the model illustrates how barriers and resources are not limited to a single level but may operate in a transversal way and how relational and structural context are associated with individual experiences. For example, the absence of systemic support (macro level) was described by caregivers in association with feelings of inadequacy (micro level), while strong familial support (meso level) and perceived personal skills were described as co-occurring with emotional burden and resilience (micro). These examples of associations are exploratory and descriptive. Figure 1 provides a visual synthesis of the model.

## Discussion

The study involved twenty-five family caregivers of individuals with dementia, exploring their characteristics, experiences and health needs, and identifying key barriers and resources to inform support interventions.

This study offers three principal contributions to dementia caregiving research in community-based settings. First, it conceptualizes caregiving as a multidimensional process shaped by structural, relational, and individual dynamics, rather than solely as burden or skill-based support. In this way it also highlights how structural healthcare gaps and relational transformation jointly shape caregivers’ experiences. Second, it empirically identifies specific resources and processes that may sustain adaptation to the role. Third, it, practically, integrates barriers and resources into a single analytic model that may guide the design of supportive interventions, by mapping both difficulties and strengths. Indeed, the findings highlight resources and elements that offer implications for the intervention’s design, sustaining the need for support pathways that recognize caregivers’ evolving and context-specific needs.

Regarding the first contribution, managing complex needs, changes in familiar and social relationships and processes of personal transformation, emerged as a central theme. These experiences were often described negatively, supporting the concept of an *‘invisible burden’*, whereby caregivers’ suffering is not always visible or acknowledged [35, 36, 37]. Additionally, caregivers reported difficulty in recognizing and accepting their emotional vulnerability, which may limit help-seeking. Partner caregivers appeared to minimize their health needs more than adult children, often normalizing stress as part of their role [38].

In the conceptual model, factors related to caregivers’ interactions with the health and social care system and environmental determinants of wellbeing were less represented, possibly due to an early stage of awareness and a lack of tools to articulate their role. Consistent with this perspective, evidence suggests that the physical and social characteristics of the neighborhood shape psychological well-being, social inclusion, and quality of life among older adults [39]. Environmental factors such as accessibility, safety, and the presence of supportive community infrastructures can enhance participation and mitigate stress, particularly in vulnerable groups such as family caregivers [40], underscoring the importance of addressing these dimensions in intervention design.

Turning to the second contribution, the analysis identified resources and processes that seem to support caregivers’ adaptation to the role. Relational dimensions emerged as another important theme in the qualitative analysis, strongly linked to caregivers’ quality of life and well-being. The interviews highlighted how changes in family and social relationships, along with shifts in reciprocity, were experienced as shaping the caregiving process. Consistently, the literature highlights how the quality of the relationship between the person with dementia and the caregiver is associated with life satisfaction and well-being for both members of the dyad [41].

Further insight into caregivers’ emotional experience emerged from the description of the sample using the WHO-5 and DASS-21 tests. The WHO-5 scores suggested a potential risk of depression, in line with emotional distress and anticipatory grief emerged from qualitative analysis.

Results also indicated that the greatest burden emerged in the strong limitation of personal and individual time, a finding that was particularly evident within the theme n.3 and the sub-themes of *loss of self* and *personal growth*, which were the most represented. This dimension of burden is further contextualized by the descriptive results of the CBI subscale S, suggesting that caregivers may perceive a loss of opportunities compared with their peers [42]. Indeed, the burden may be further aggravated when the complexity of caregiving is not accompanied by institutional training and support pathways. In this regard, the descriptive data on HL align with qualitative findings and underline the importance of considering caregiver HL within geriatric assessment, as well as the potential values of interventions aimed at strengthening it [43]. In this context, HL may be viewed as an important factor associated with caregiver empowerment, and, in turn, caregiving processes and the quality of care, as suggested by existing literature.

Another aspect that seems to be related to the process of adaptation is the quality of the relationship prior to the onset of dementia [44, 45, 46].

Our findings also emphasized the importance of coping strategies that may be associated with the perception of stress and depression as well as quality of life [47]. Caregivers mainly using problem-focused strategies, such as active planning and management of daily challenges, and positive emotional strategies, such as optimism and acceptance, tended to experience better psychological well-being. Conversely, dysfunctional coping strategies, such as self-blame, denial, and excessive worry, appear to be associated with negative effects on caregivers’ emotional state [48]. In accordance with this evidence, other studies highlighted how a greater sense of self-efficacy and self-esteem is associated with better personal health and a greater propensity to actively seek help [49, 50, 51].

The third contribution concerns the practical implications of these findings. The barriers and resources analysis model we developed emphasized that promoting caregiver wellbeing may require attention not only to individual support but also to relational and institutional factors, recognizing the interconnections between personal experiences, family dynamics, and organizational aspects. We suggest an integrated approach for defining supportive interventions for informal caregivers, organized across the three levels of our conceptual model, each providing specific strategies to address the evolving needs of caregivers: the micro or individual level – including psychological support, training, empowerment, psychoeducational programs, and self-help groups; the meso or community level – involving the strengthening of family and social networks through improved communication and the active engagement of caregivers in care planning; the macro or systemic level – through the formal recognition of caregivers’ role, better access to care, and the provision of dedicated services in primary care centres.

The proposed model also promotes a strengths-based approach that values resilience in the caregiving experience [52]. From this perspective, caregivers should not be recognized only as bearers of needs and vulnerabilities, but also as an integral part of healthcare and considered partners of healthcare professionals [53]. Accordingly, it would be desirable to provide them with tools, information, and adequate support to better fulfil their role. For example, educational experiences based on *problem solving and mindfulness* have been associated with improvements in strengthening self-efficacy and promoting adaptive coping strategies [54, 55, 56]. Caregiving may be more likely to be perceived as a positive experience when self-efficacy is high [57]. This element, as also shown in our results, may foster personal growth and may help reduce feelings of inadequacy or meaninglessness. Additionally, educational programs integrating psychotherapeutic components, counselling and mindfulness-based interventions may be effective in reducing depressive symptoms among caregivers [52]. For these reasons, interventions aimed at enhancing self-efficacy and promoting active coping strategies may help reduce caregivers’ burden. Examples include psychological approaches that provide emotional, informational, and instrumental support as well as mindfulness interventions [58], although more data are needed to assess their long-term impact. Moreover, support groups and community-based strategies have been associated with positive outcomes for caregivers’ health, and they may also strengthen the perceived meaning of self and care, a crucial element in building a sense of purpose in caregiving [59, 60]. Structural interventions - such as integrating caregivers into the multidisciplinary care team and ensuring continuity of care - may help address the feeling of invisibility and enhance trust in care pathways [61].

In this regard, Stephan et al. [62] suggest the elements useful to improve collaboration between healthcare professionals and caregivers, including simplified access to services and the presence of a permanent contact person for caregivers—such as a case manager. This process can also benefit from training healthcare professionals in interprofessional collaboration [63] and empathetic communication and reinforcing the central role of family in caregiving. Such skills may play a crucial role in improving relationships, promoting a collaborative environment, and reducing fragmentation of care [64].

### Limitations and strengths

The study has some limitations but also strengths that should be acknowledged. Participants were recruited through convenience sampling in a single-site setting and if they had expressed early interest in participating in the planned group-based support intervention. This recruitment strategy may have led to the inclusion of a particularly motivated subgroup of caregivers and may limit the transferability of the findings to other contexts. In addition, although the sample size was appropriate for the qualitative aims of the study, it does not allow for quantitative inferential analyses

. While the study was open to informal caregivers, the final sample consisted exclusively of family caregivers; therefore, the findings primarily reflect family-based caregiving experiences, in which responsibility, guilt, and family dynamics may be particularly salient. The cross-sectional design and qualitative nature of the study do not allow conclusions to be drawn about changes in caregiving experiences over time, nor do they allow for causal inferences. In addition, interviews were not audio-recorded. Although a detailed written transcription was produced in real time, jointly reviewed by two researchers, and checked with participants, the resulting data do not constitute verbatim transcripts. This may have limited the capture of nuances such as wording, pauses, tone, and emphasis. As a result, the written transcripts may have been shaped by researchers’ interpretative and selective decisions during data capture. However, as described in the methods section, procedures were adopted to enhance accuracy and minimize potential bias in data collection and analysis.

Despite these limitations, the study also presents important strengths. The diversity of the caregivers’ profiles included - particularly in terms of relationship with the care recipient (e.g., spouse, son/daughter), cohabitation status, and caregiving duration - enabled the inclusion of a diversified set of experiences and allowed for a more comprehensive understanding of caregiving as a multidimensional process.

## Conclusion

This study highlights the complex burden experienced by family caregivers of individuals with dementia. Limited personal time, perceived loss of opportunities, and the cumulative effects of emotional and relational burden were associated with significant caregiver burden and negative health impacts. The findings suggest the importance of addressing HL, coping strategies, and resilience, as well as the quality of relationships. Moreover, a better understanding of these dimensions and of caregivers’ experiences is essential to inform the design of supportive strategies in primary care and community settings.

## Supplementary Information

Below is the link to the electronic supplementary material.

Supplementary Material 1

## Data Availability

Aggregated quantitative and qualitative data that support the findings may be shared from the corresponding author upon reasonable request. Due to the sensitive nature of qualitative data, the data are not publicly available.
