# Supplementary material for: Needs, resources, and barriers among family caregivers of people with dementia: a qualitative study
Source: Aging Clin Exp Res. 2026 Jul 3;38(1):196. doi: 10.1007/s40520-026-03436-x (PMC13601077; doi:10.1007/s40520-026-03436-x)
Supplement: Supplementary file 1 [file 40520_2026_3436_MOESM1_ESM.docx]

**Supplementary file 2**. Example of coding tree.

| **Example quote** | **Initial code** | **Original sub-theme**  **(Meaning making)** | **New/adapted sub-theme** | **Theme** |
| --- | --- | --- | --- | --- |
| “She depends on me for everything” | Constant caregiving presence | Complicated, progressive, and unresponsive demands | Managing complex needs | Complicated demands and healthcare interactions |
| “Everything is exhausting” | Resource depletion | Complicated, progressive, and unresponsive demands | Managing complex needs | Complicated demands and healthcare interactions |
| “Institutions don’t help her” | Lack of institutional support |  | Interactions with services | Complicated demands and healthcare interactions |
| “My brothers don’t realize the burden” | Unequal burden distribution | Dilemmatic familial relationships | Changes in familiar and social relationships | Family and relational dynamics |
| “He is no longer himself” | Loss of reciprocity | Ambivalence in dyadic relationships. | Shift in reciprocity and redefinition of role | Family and relational dynamics |
| “I don’t do anything anymore” | Identity erosion | Balance between caregiving and personal expectation | Identity changes and personal transformation | Coping strategies |
| “That month shaped me” | Personal growth | Perception of loss and growth | Identity changes and personal transformation | Coping strategies |
